# Effects of high-intensity interval exercise on cardiac troponin elevation when comparing with moderate-intensity continuous exercise: a systematic review and meta-analysis

**DOI:** 10.7717/peerj.14508

**Published:** 2023-01-11

**Authors:** Shuoqi Li, Shazlin Shaharudin, Rafel Cirer-Sastre, Feifei Li, Faizal Abdul Manaf, Mohd Faiz Mohd Shukri

**Affiliations:** 1 School of Sports Science, Nantong University, Nantong, China; 2 Exercise & Sports Science Programme, School of Health Sciences, Universiti Sains Malaysia, Kota Bharu, Malaysia; 3 National Institute of Physical Education of Catalonia; Research Group Human Movement, Universitat de Lleida, Lleida, Spain; 4 Center for Health and Exercise Science Research; Department of Sport, Physical Education and Health, Hong Kong Baptist University, Hong Kong, China; 5 Defence Fitness Academy, Universiti Pertahanan Nasional Malaysia, Kuala Lumpur, Malaysia; 6 Department of Emergency Medicine, School of Medical Sciences, Universiti Sains Malaysia, Kota Bharu, Malaysia; 7 Hospital Universiti Sains Malaysia, Health Campus, Universiti Sains Malaysia, Kota Bharu, Malaysia

**Keywords:** Intermittent exercise, Human health, Exercise physiology, Sports medicine

## Abstract

**Background:**

This systematic review and meta-analysis aimed to compare the effects of high-intensity interval exercise (HIIE) with different recovery modes versus moderate-intensity continuous exercise (MICE) on cardiac troponin (cTn) elevation.

**Methodology:**

A literature search was conducted in four databases: Scopus, PubMed, EBSCO and Web of Science from January 2010 to June 2022. The articles were screened, evaluated for quality before data were extracted. The review protocol was registered at PROSPERO (CRD42021245649). Standardized mean differences (SMD) of peak cTn were analyzed with a 95% confidence interval (95% CI) using Revman 5.4 software.

**Results:**

Six studies satisfied the inclusion criteria with a total of 92 and 79 participants for HIIE and MICE, respectively. Overall, there was no significant difference between HIIE and MICE in the elevation of cardiac troponin T (SMD: 0.41 [95% CI [−0.21, 1.03]], *p* = 0.20, *I*^2^ = 77%, *p* for heterogeneity <0.01). In subgroup analysis, HIIE with passive recovery elicits greater release of cardiac troponin T than MICE (SMD: 0.85 [95% CI [0.44, 1.27]], *p* < 0.01, *I*^2^ = 32%, *p* for heterogeneity = 0.22). Changes of cardiac troponin T (SMD: 0.41 [95% CI [−0.21, 1.03]], *p* = 0.20, *I*^2^ = 77%, *p* for heterogeneity < 0.01) after HIIE with active recovery were not significantly different from those of MICE.

**Conclusions:**

There was no significant difference between HIIE and MICE in the elevation of cardiac troponin T. However, HIIE with passive recovery elicited more cardiac troponin T elevation than MICE, which should be considered when developing exercise programs.

## Introduction

The overall risk of sudden death from exercise ranges from 0.1 to 38 per 100,000 person-years (Drezner, 2000), which is considered relatively low. However, the risk of cardiac related adverse events such as sudden cardiac death during high-intensity exercise still exists (Lippi, Favaloro & Sanchis-Gomar, 2018). High-intensity interval exercise (HIIE) refers to a type of interval exercise in which active or passive recovery periods are interspersed with high-intensity exercise (Coletta et al., 2021). In comparison to moderate-intensity continuous exercise (MICE), HIIE is more effective in improving participants’ aerobic capacity, oxidative stress, visceral fat, and insulin sensitivity (Devin et al., 2018; Coletta et al., 2019; Maturana et al., 2020; Wu et al., 2021). Yet, due to the heavy cardiopulmonary load during HIIE, its cardiovascular risk cannot be overlooked despite its benefits (Thompson et al., 2007).

Cardiac troponin is the gold standard for clinical diagnosis of myocardial injury (Thygesen et al., 2019), and may be related to the heart risk after exercise (Kleiven et al., 2020). With the advancement of assay generation and the lower limit of detection in cardiac troponin assays, it has become an accurate and reliable marker in the screening of cardiac damage. In clinical settings, cardiac troponin increases sharply after irreversible damage, which is maintained for a long time (Park et al., 2017). Nevertheless, the increase in cardiac troponin induced by exercise was different in terms of hemodynamic changes (Li et al., 2021a; Eijsvogels et al., 2015; Cirer-Sastre et al., 2021). Most studies have shown that the decrease from the peak occurred after 3–6 h following exercise (Park et al., 2017; Eijsvogels et al., 2014; Hosseini et al., 2018; Eijsvogels et al., 2015; Chan-Dewar et al., 2013). On the other hand, the discrepancy interfered with the accuracy of clinical diagnosis of myocardial infarction when using cardiac troponin. Therefore, it is crucial to explore the changes in cardiac troponin after various types of exercise.

Overall intensity, duration, and exercise types are factors that affected the level of cardiac troponin after exercise (Li et al., 2021a; Takahashi & Miyamoto, 1998; Martínez-Navarro et al., 2020; Richardson et al., 2018). The differences were evident particularly in the comparison of exercise intensity such as between HIIE and MICE (Weippert et al., 2016; Zhang et al., 2019). In the same vein, some studies have shown that the increase in cardiac troponin after HIIE was greater than that of MICE (Weippert et al., 2016; Nie et al., 2020), which contradicts the findings from another study (Ranjbar et al., 2017). HIIE’s recovery period could be either active or passive depending on whether participants having active or passive rest during intervals. Active recovery involved low-intensity exercise during the interval while passive recovery involved being stationary during intervals (Madueno et al., 2019). A recent study reported that the duration of elevated heart rate is an important predictor of the increased level of exercise-induced cardiac troponin  (Bjørkavoll-Bergseth et al., 2020). Furthermore, active recovery might be more effective in eliminating lactic acid accumulation (Miladi et al., 2011), improving heart rate recovery (Takahashi et al., 2001), increasing venous blood return (Takahashi & Miyamoto, 1998) and maintaining higher stroke volume (Takahashi & Miyamoto, 1998) than passive recovery. The release of cardiac troponin may be affected by the myocardial blood supply and cardiac mechanical stress.

Shave et al. (2007) conducted a meta-analysis regarding the effects of exercise on cTn and found that compared with other athletes, endurance athletes released more cTn after exercise. A review by Cirer-Sastre et al. (2019) also indicated that the peak value of cTn might be related to the amount of exercise and the intensity of exercise. However, to the best of our knowledge, currently there are no specific review on the effects of HIIE *versus* MICE on post-exercise cTn elevation. Therefore, this review aimed to explore the effects of recovery mode in HIIE *versus* MICE on the level of cardiac troponin after exercise. This information can help clinicians to estimate the outcomes of cardiac troponin after exercise, reduce misdiagnosis caused by exercise-induced cardiac troponin elevation, and help to establish safe HIIE exercise prescriptions.

## Methodology

### Data sources and study selection

The literature search with restricted timeline from January 2010 to June 2022 was performed using four electronic databases namely Web of Science, Scopus, PubMed and EBSCO. The last retrieval date was June 23, 2022. The search terms included “Interval”, “Intermittent”, “Training”, “Train”, “Exercise”, “cTn”, and “Troponin”. Information experts were responsible for the search strategy as described in Appendix A and the corresponding results. Two investigators (SL, SS) individually screened the titles and abstracts retrieved from the databases. Thereafter, full articles were reviewed based on inclusion and exclusion criteria. Finally, two investigators (SL, SS) carried out the data extraction. If there was any discrepancy or disagreement between the two investigators, a consensus was reached by inviting another investigator (MFMS). Upon reconfirming the included studies, their references were also searched manually.

**Figure 1:**
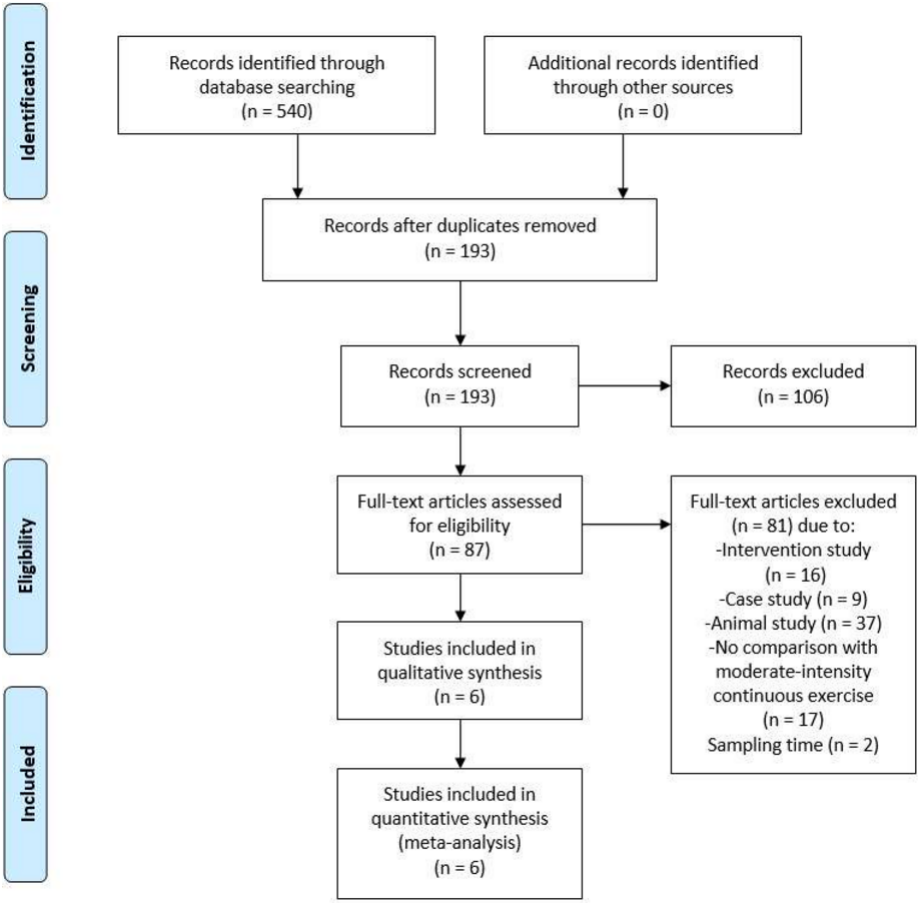
Preferred Reporting Items for Systematic Reviews and Meta-Analysis (PRISMA) flow diagram of search results.

The review protocol was registered at PROSPERO international prospective register of systematic reviews (CRD42021245649) on 28 April 2021 before performing formal screening. The Preferred Reporting Items for Systematic Reviews and Meta-Analysis (PRISMA) guidelines were followed in identifying the research articles and writing the review. The article selection process is presented in Fig. 1.

### Inclusion and exclusion criteria

The inclusion criteria were based on the following: (1) Population: Participants over the age of 18; (2) Intervention: Intervention group involved HIIE; (3) Comparison: Control group involved MICE; (4) Outcomes: Troponin T were measured at baseline and within the 1st–4th hour post-exercise; Troponin concentrations were reported as mean (standard deviation) and/or median (interquartile range); (5) Study: Randomized controlled trials and cross-over studies; (6) Other: Full-text papers in English. Abstracts, conference proceedings, or poster presentations were not considered in this study.

### Quality assessment

Cochrane risk of bias assessment tool was used to evaluate the methodological quality of the included studies (Higgins et al., 2019). This tool assesses random sequence generation, allocation concealment, blinding of participants and personnel, blinding of outcome assessment, incomplete outcome data, selective reporting, and other biases in the included studies (Cirer-Sastre et al., 2019). Items were scored as “yes”, “no”, or “unclear” to describe the quality of the included studies. Two reviewers (LS and SS) assessed the risk of bias independently, and disputes were solved by discussion with a third author (MFMS).

### Data extraction

Details from each included study were extracted and tabulated, such as study population, age, sample size and gender, exercise program, equipment, intensity, duration, heart rate, baseline, and peak values of relevant cardiac biomarkers, and when the peak value appeared post-exercise (Tables 1 and 2). If two HIIE programs met the inclusion criteria in one article, data of the two HIIE programs were combined according to the method recommended by the Cochrane manual  (Higgins et al., 2019), and the MICE program remained unchanged. This combination was achieved by adding the sample size of the two HIIE groups and averaging the mean and standard deviation. If the study reported two or more cardiac troponin values after exercise, the highest one was selected as the peak value.

**Table 1:** Overview of the included studies.

Author	Population	Cardiac health status	Age (y)	Sample size	Exercise program	Baseline value (ng/L)	Peak value post-exercise (ng/L)	Timing of peak value (h)
HIIE with active recovery mode								
Li 2020 (Li et al., 2020)	Athlete	No CVD by self-report	23.5 ± 5.5	11 Male, 1 Female	HIIE	8.3 ± 12.6	40.4 ± 27.6	4
					MICE	6.9 ± 8.1	50.0 ± 63.2	
Ranjbar 2017 (Ranjbar et al., 2017)	Health	Normal ECG	22.3 ± 1.9	11 Male	HIIE	4.3 ± 0.8	5.5 ± 0.9	1
					MICE	4.9 ± 0.5	8.0 ± 2.5	
HIIE with passive recovery mode								
Nie 2018 (Nie et al., 2018)	Overweight	No CVD by self-report	21.0 ± 1.1	16 Female	HIIE	2.9 ± 1.0	8.6 ± 6.0	2–4
			20.9 ± 1.6	14 Female	MICE	3.2 ± 1.1	5.5 ± 3.5	
Nie 2020 (Nie et al., 2020)	Health	No CVD by self-report	21.5 ± 2.6	17 Female	HIIE	3.1 ± 1.8	10.4 ± 6.1	3–4
					MICE	3.4 ± 1.4	6.5 ± 4.3	
Weippert 2016 (Weippert et al., 2016)	Health	Normal ECG; LVEF=64.3 ± 9.9%	26.2 ± 2.9	13 Male	HIIE	5.0 ± 1.8	10.0 ± 4.6	4
					MICE	5.0 ± 1.2	5.7 ± 1.6	
Zhang 2019 (Zhang et al., 2019)	Overweight	No CVD by self-report	18–25	12 Female	HIIE	1.8 ± 0.9	9.3 ± 8.9	3–4
									11 Female	HIIE	2.1 ± 1.1	10.6 ± 10.6	
				12 Female	MICE	2.0 ± 1.2	6.9 ± 7.2	

**Notes.**

HIIE high-intensity interval exercise MICE moderate-intensity continuous exercise CVD cardiovascular diseases ECG electrocardiogram LVEF left ventricular ejection fraction hs-cTnT high-sensitivity cardiac troponin T, assay manufactured by Roche, detection limit 3 ng/L and 99th upper reference limit 14 ng/L

**Table 2:** Exercise program of the included studies.

Author	Equipment	Exercise program	Intensity	Duration (min)	Averaged HR (beat/min)	Exercise HR (beat/min)	Recovery HR (beat/min)
HIIE with active recovery mode							
Li 2020 (Li et al., 2020)	Treadmill	HIIE	2 min 90% VO_2max_: 2 min 50% VO_2max_	92	160.0 ± 12.0	176.0 ± 12.0	145.0 ± 13.0
		MICE	70% VO_2max_	92	162.0 ± 11.0	–	–
Ranjbar 2017 (Ranjbar et al., 2017)	Treadmill	HIIE	1 min 80% HRr: 2 min 50% HRr	40	107.6 ± 10.8	NA	NA
		MICE	60% HRr	40	101.7 ± 5.7	–	–
HIIE with passive recovery mode							
Nie 2018 (Nie et al., 2018)	Ergometer	HIIE	4 min 90% VO_2max_: 3 min Rest	30 ± 4	NA	151.0 ± 17.0	NA
		MICE	60% VO_2max_	51 ± 5	146.0 ± 19.0	–	–
Nie 2020 (Nie et al., 2020)	Ergometer	HIIE	4 min 90% VO_2max_: 3 min Rest	46 ± 2	NA	155.0 ± 3.0	NA
		MICE	60% VO_2max_	85 ± 4	137.0 ± 4.0	–	–
Weippert 2016 (Weippert et al., 2016)	Treadmill	HIIE	30 s 90% HR_max_: 15 s Rest	21	NA	174.1 ± 9.8	NA
		MICE	70% HR_max_	60	136.3 ± 6.2	–	–
Zhang 2019 (Zhang et al., 2019)	Ergometer	HIIE	4 min 90% VO_2max_: 3 min Rest	28 ± 3	NA	157.0 ± 9.0	NA
				HIIE	1 min 120% VO_2max_: 1.5 min Rest	21 ± 2	NA	148.0 ± 11.0	NA
		MICE	60% VO_2max_	63 ± 12	140.0 ± 12.0	–	–

**Notes.**

HIIE high-intensity interval exercise MICE moderate-intensity continuous exercise VO_2max_ maximal oxygen consumption HR heart rate HR_max_ maximum heart rate HRr heart rate reserve NA not available - not applicable

### Data analysis

All the data were entered into the Review Manager (Version 5.4.1, Copenhagen: The Nordic Cochrane Center, The Cochrane Collaboration, 2020) to perform meta-analysis. The outcomes of the included studies consisted of continuous variables but the test methods were different. Therefore, standardized mean difference (SMD) was chosen as the index of effect scale. When outcomes provided in the studies were reported as median (range), it was converted to the average (standard deviation) based on the method described by Hozo, Djulbegovic & Hozo (2005) The heterogeneity between studies was tested using the *I*^2^ and Tau^2^ statistics. Accordingly, the smaller the Tau^2^, the smaller the heterogeneity between studies. Also, no heterogeneity between the studies if total *I*^2^ was less than 50%. Thus, a fixed effect model was applied for analysis. In contrast, heterogeneity between the studies existed if total *I*^2^ was equal to or greater than 50%. Hence, a random effect model must be applied for analysis (Li et al., 2021b). To further determine the heterogeneity, sub-group analysis was conducted. Publication bias was assessed with funnel plot, while SMD was evaluated with Forest plot. The measure of uncertainty was calculated as 95% confidence intervals (95% CI).

## Results

### Eligibility of studies

Tables 1 and 2 show the details of the eligible studies. Despite a rigorous search, only two randomized control trials (Zhang et al., 2019; Nie et al., 2018) and four cross-over design (Weippert et al., 2016; Nie et al., 2020; Ranjbar et al., 2017; Li et al., 2020) studies met the criteria of the review. Protocols for all the included studies were ethically approved by their respective institutions. Two investigators completed the calibration exercise to ensure that their assessments were consistent. Cohen kappa coefficient between the two investigators was *k* = 0.93. The six studies included 35 men and 83 women, with 92 participants for the HIIE and 79 participants for the MICE. During the recovery of HIIE, two studies (Ranjbar et al., 2017; Li et al., 2020) used active recovery, and four studies (Weippert et al., 2016; Zhang et al., 2019; Nie et al., 2020; Nie et al., 2018) used passive recovery. One study (Nie et al., 2020) reported two HIIE programs where the two programs were combined into one data set. The assay method of cardiac troponin in all studies was the 5th generation high-sensitivity immunoassay.

### Quality assessment and analysis of publication bias

**Figure 2:**
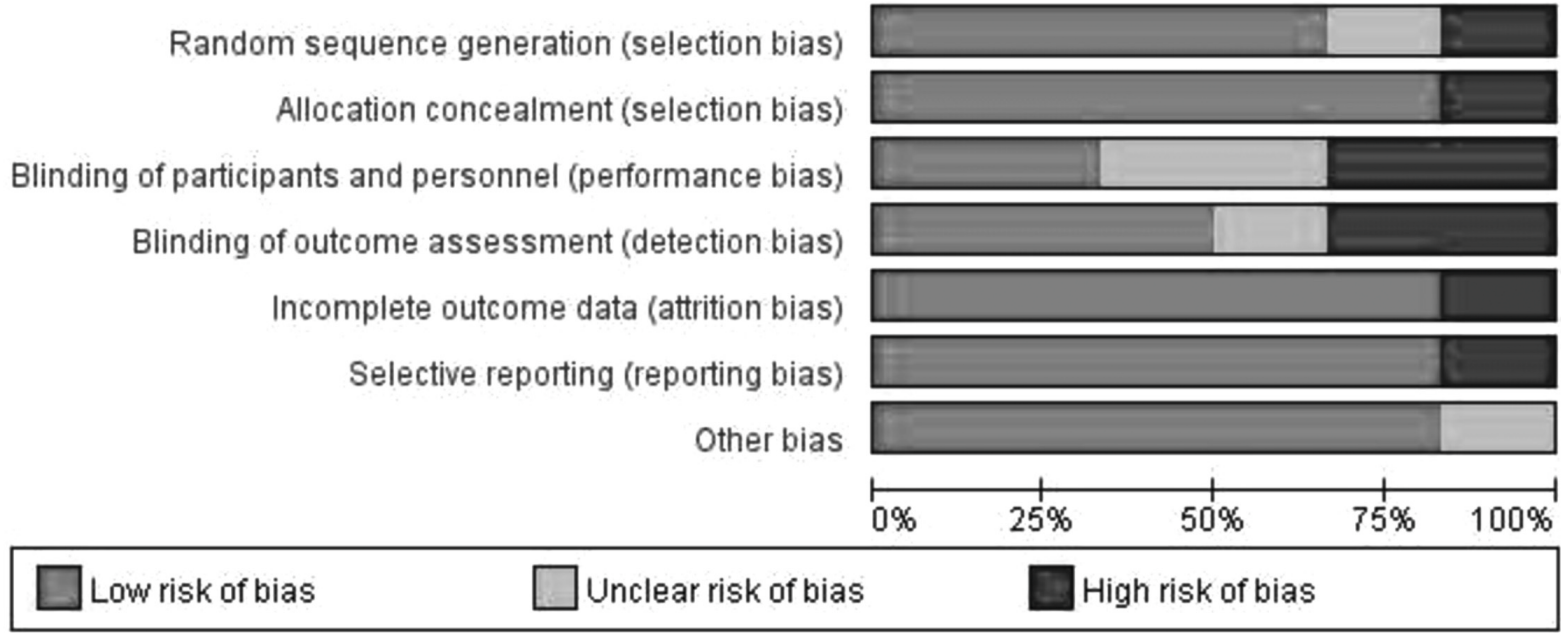
Analysis of risk of bias according to Cochrane Collaboration guideline.

Figure 2 shows an assessment of the methodological quality and potential risk of bias of the included studies. The results showed that the overall quality of the included studies was relatively high. High, unclear, and low risk of bias accounted for 11.9%, 11.9%, and 76.2%, respectively. Figure 2 shows the risk of bias for each included study.

**Figure 3:**
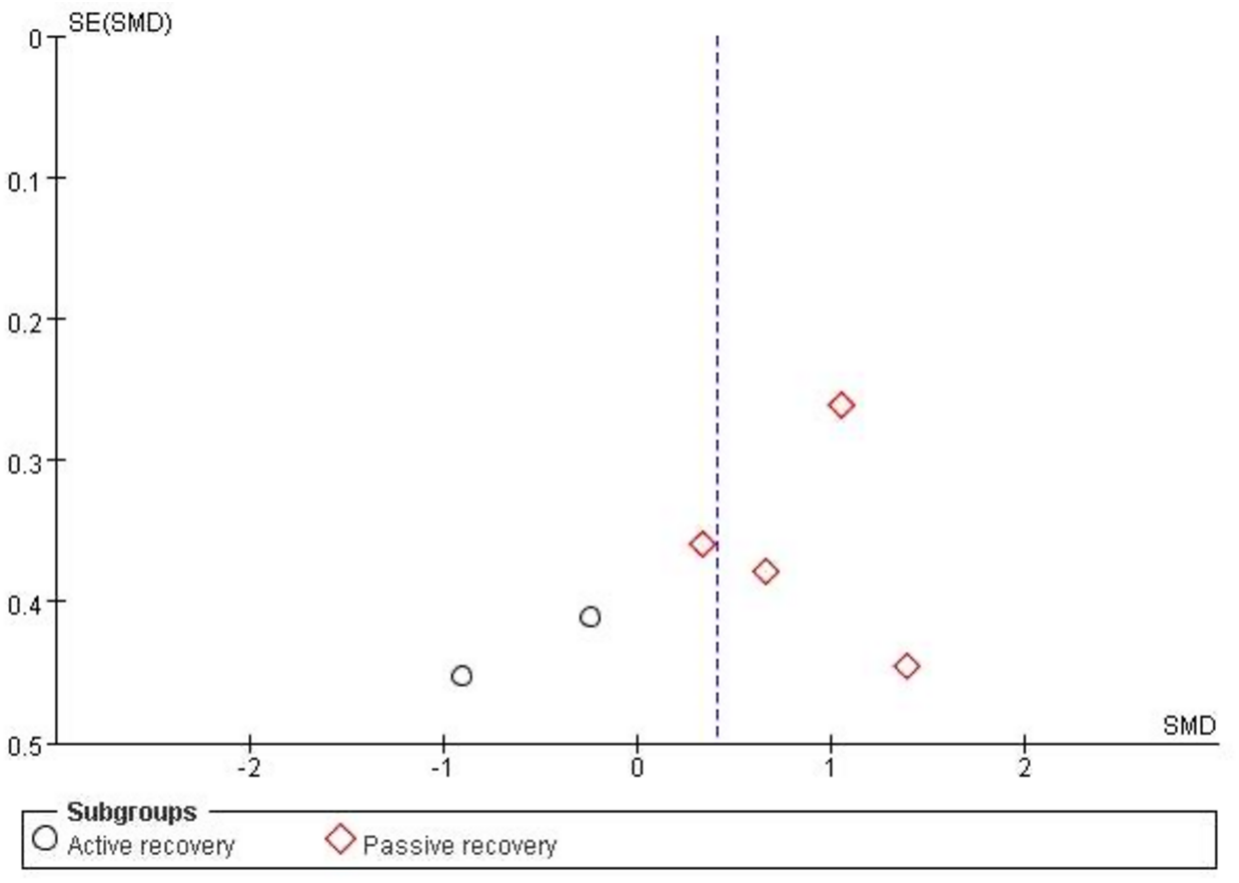
Funnel plot of publication bias for cardiac troponin in the HIIE *vs* MICE model.

The funnel plot was used to analyze the publication bias of the included studies. As the total number of the included studies was close to the minimum requirement of using a funnel plot (Lu et al., 2020), publication bias can be reflected to a certain degree. Figure 3 shows that both figures formed a left–right symmetrical distribution, suggesting a low chance of publication bias.

### Sensitivity analysis

Sensitivity analysis was conducted by modifying the type of analysis model, altering the effect size, and excluding individual studies. After the sensitivity analysis, there was no significant change in the results of each sub-group in the meta-analysis; thus, the results were reliable.

### Quantitative synthesis

**Figure 4:**
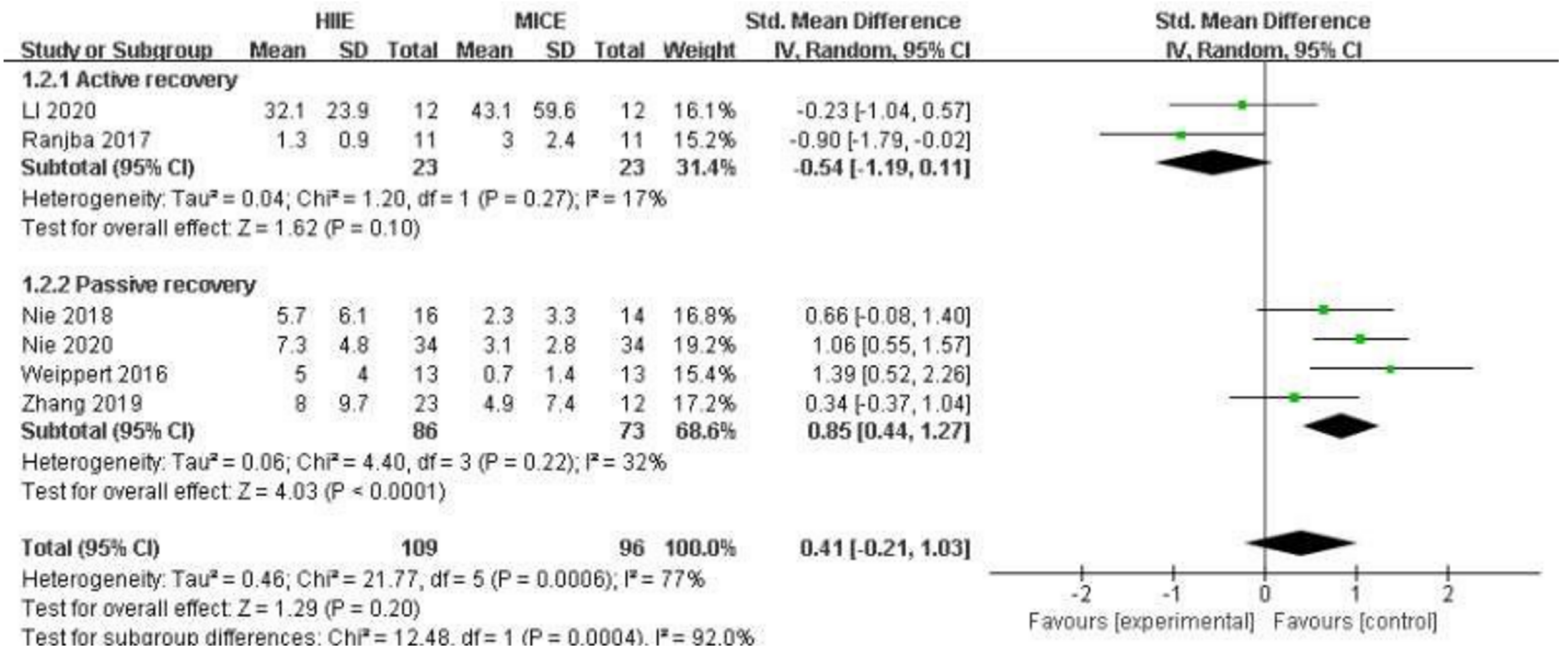
Forest plot illustrating the effects of HIIE *vs* MICE on cardiac troponin. HIIE high-intensity interval exercise; MICE moderate-intensity continuous exercise.

Six of the included studies compared the effects of HIIE (*n* = 109) and MICE (*n* = 96) on cardiac troponin T (Weippert et al., 2016; Zhang et al., 2019; Nie et al., 2020; Ranjbar et al., 2017; Nie et al., 2018; Li et al., 2020). Overall, there was no significant difference between HIIE and MICE in the elevation of cardiac troponin T (SMD: 0.41 [95% CI [−0.21, 1.03]], *p* = 0.20, *I*^2^ = 77%, *p* for heterogeneity < 0.01) (Fig. 4). In the sub-group analysis, the increase of cardiac troponin T after HIIE with passive recovery was significantly higher than that of MICE (SMD: 0.85 [95% CI [0.44, 1.27]], *p* < 0.01, *I*^2^ = 32%, *p* for heterogeneity = 0.22). Details are shown in Fig. 4.

## Discussion

The present results showed that the release of cardiac troponin is unclear by different types of exercises, thus more studies are needed to clarify its effects. Interestingly, sub-group analysis revealed that HIIE recovery modes, *i.e.,* active and passive, *versus* MICE may lead to differences in the release of cardiac troponin after exercise. This novel finding proposed a modifiable factor to exercise-induced cardiac troponin elevation. No research has directly compared the effects of HIIE especially with multiple recovery modes and MICE on cardiac troponin. Therefore, when formulating HIIE exercise prescriptions and conducting research, the impact of recovery modes on the release of cardiac troponin must be considered.

According to the current results, most studies related to the effects of HIIE and MICE on cardiac troponin need to match the amount of exercise, such as the averaged intensity and time (Tschakert et al., 2016) or the total work (Zhang et al., 2019; Nie et al., 2020). This probably due to HIIE with passive recovery resulted in more time spent on exercising than with active recovery. As shown by a previous study, longer exercise duration at high intensity resulted in increased cardiac troponin (Bjørkavoll-Bergseth et al., 2020). Hence, the duration of exercising at high-intensity may be the main factor that influences the release of cardiac troponin (Li et al., 2021a).

In the study of Yamagishi & Babraj (2019), a 2-week sprint interval training of 30s followed by a 4-min recovery was performed for three sessions per week. The active recovery group exercised at 40% VO_2 peak_ during rest intervals, whereas the passive recovery group rested. There was no significant difference in the VO_2 max_ between the two groups after training for two weeks. In contrast, Abderrahmane et al. (2013) found that after seven weeks of HIIE training with the same amount of exercise, only the active recovery group significantly improved VO_2 max_ and maximum aerobic speed. The present results indicated that active recovery led to the release of less cardiac troponin after exercise, which might be related to the improvement of aerobic capacity.

In addition, improvement of acid–base balance (Miladi et al., 2011; Siegler et al., 2006), maintenance of stroke volume (Takahashi & Miyamoto, 1998), oxygen uptake (Miladi et al., 2011; Takahashi et al., 2001; Takahashi & Miyamoto, 1998), and venous return (Takahashi & Miyamoto, 1998) could contribute to the exercise-induced cardiac troponin level. In the study conducted by Nalbandian, Radak & Takeda (2017), 17 athletes randomly performed two HIIE sessions. The exercise program consisted of three sets of 30s exercise at an intensity of 90% maximum power (P_max_) with a 4-min active or passive recovery between each bout. The outcomes revealed that the rate of lactate removal during active recovery was significantly higher than that during passive recovery. Furthermore, Takahashi & Miyamoto (1998) stated that a combination of 7-min active recovery and 3-min passive recovery could reduce the heart rate and maintain the stroke volume than 10-min passive recovery only.

On the contrary, two studies have shown that stroke volume was maintained at a higher level during active recovery than passive recovery (Takahashi & Miyamoto, 1998; Miladi et al., 2011). This might lead to an increase in blood flow in the coronary artery and thus improve the blood supply of the myocardium. Similarly, a high level of oxygen uptake during active recovery could also facilitate early oxygen contribution to promote the removal of body metabolites and fatigue recovery (Kriel et al., 2016). Therefore, active recovery might be able to change the level of cardiac troponin by changing physiological responses such as acid–base balance and myocardial blood supply.

There is no clear mechanism of the increase in cardiac troponin caused by exercise. Permeability alterations of the myocardial cell membrane remain the widely recognized mechanism. High-intensity exercise might be able to change the mechanical stress (Clarke et al., 1995; McNeil & Khakee, 1992), oxidative stress (Nie et al., 2010; Nie et al., 2016) and acid–base balance (Sahlin, 1980) of the cardiomyocytes to induce temporary destruction of the myocardial cell membrane, thereby increasing the permeability of the cell membrane. Starnberg et al. (2014) conducted an *in vitro* study on human heart tissue and found that the increased permeability of myocardial muscle membrane might promote the temporary leakage of cytoplasmic cardiac troponin, including free radical-mediated damage (Shave et al., 2010). Similar findings were reported in the study of Nie et al. (2010), involving the use of rat models. In the study, 15 rats were divided into four groups and tested before 3 h of swimming exercise, immediately after exercise, and at 2 h and 24 h post-exercise. The results showed that cardiac troponin T increased significantly immediately and at 2 h after exercise. This increment was related to the increased of lipid peroxidation in the rat’s heart. It indicated that oxidative stress might be an important cause of cell membrane damage and cardiac troponin leakage. On the other hand, antioxidants such as Vitamin E appear to protect muscle cells by reducing oxidative stress (Hickman et al., 2010) and rates of lipid peroxidation (Bleier et al., 1998). A study showed that supplementing with vitamin E following a brief bout of vigorous exercise still resulted in a substantial increase in cardiac troponin, but the level of significance was reduced when compared to the group that did not receive vitamin E supplementation (Remppis, 1995). Thus, dietary and enzymatic antioxidants may play a role in modifying the level of cardiac troponin.

Exercise-induced cardiac troponin might be transported through membranous blebs (Schwartz et al., 1984). Cardiac troponin mainly bonded to myofibrils, whereas the unbound cardiac troponin T and I in the cytoplasm were about 5–8%(Bleier et al., 1998). This made it possible for cytoplasmic cardiac troponin to enter the peripheral blood after exercise. A study indicated that temporary ischemia and reoxygenation could initiate the instantaneous release of cardiac troponin (Remppis, 1995). However, if the period of ischemia was extended before reoxygenation, troponin release was greater and more prolonged, which may be related to the production of membranous blebs after cell ischemia. This view corroborates with the present result that the release of cardiac troponin was greater when the ischemic time was longer during passive recovery. Similarly, the study by Schwartz et al. (1984) demonstrated that the cultured cardiomyocytes developed membrane vesicles and released cytoplasmic enzymes in the absence of oxygen without cell necrosis. These findings suggested that the release of cardiac troponin after exercise might not come from irreversible damage to cardiomyocytes. Nevertheless, most of the current indications on these pathways were ancillary and there was no direct evidence from human studies.

### Study limitations

There are data paucity on the effects of HIIE on cardiac troponin and most of the available studies employed distinct exercise’s set, intensity and duration. Secondly, after subgroup analysis, the sample size of each subgroup is very small. There are differences in the baseline measurements due to the different exercise ability of participants, thus the results of subgroup analysis should be treated with caution. In addition, long-term intervention may affect the baseline level of cardiac troponin, however the studies included in this study are all acute studies, so the baseline level was not compared. Future studies with large sample size are needed to strengthen the evidence.

Furthermore, due to the varied methodologies (*i.e.,* the timing of blood sampling and matching methods between HIIE and MICE) and data processing employed in the included studies, we applied the standardized mean difference instead of the mean difference to compare the effect size. Moreover, the current studies have not observed the recovery of cardiac troponin after exercise, which limits the interpretation of the results. Future research is recommended to investigate the effects and mechanisms of different exercise recovery methods on heart function. Finally, only articles written and published in English were included in this review.

## Conclusions

Exercise recovery mode may potentially affect the release of cardiac troponin after exercise. HIIE with passive recovery induced more cardiac troponin elevation after exercise compared to MICE. In clinical practice, this finding offers a valuable insight in the evaluation of raised cardiac troponins in patients who presented with angina equivalence symptom especially following exercise. This is particularly important for an acute care physician to differentiate a benign cause of raised cardiac troponin from myocardial infarction. Therefore, cardiac troponin values would offer a more accurate diagnostic value and should be considered, in addition to the usual clinical evaluation and electrocardiogram.

##  Supplemental Information

10.7717/peerj.14508/supp-1 Supplemental Information 1 PRISMA checklist

10.7717/peerj.14508/supp-2 Supplemental Information 2 Search strategy

10.7717/peerj.14508/supp-3 Supplemental Information 3 Rationale and differences with previous reviews

10.7717/peerj.14508/supp-4 Supplemental Information 4 The date of formal screening

## Additional Information and Declarations

### Data Availability

The following information was supplied regarding data availability: The search strategy is available in the Supplemental File.
