# Supplementary material for: Effects of high-intensity interval exercise on cardiac troponin elevation when comparing with moderate-intensity continuous exercise: a systematic review and meta-analysis
Source: PeerJ. 2023 Jan 11;11:e14508. doi: 10.7717/peerj.14508 (PMC9840388; doi:10.7717/peerj.14508)
Supplement: Supplemental Information 2 [file peerj-11-14508-s002.docx]

**Appendix A** Search strategy

| Databases | Search strategy | Result |
| --- | --- | --- |
| Scopus | #1: Title-Abs-Key ("Interval " or "Intermittent")  #2: Title-Abs-Key ("Training" or "Train" or "Exercise")  #3: Title-Abs-Key ("cTn" or "Troponin")  #4: #1 and #2 and #3  Limiters - Published Date: 20100101-20220623 | 942,008  1,324,943  43,973  249 |
| Pubmed | #1: [Title/Abstract] "Interval " or "Intermittent"  #2: [Title/Abstract] "Training" or "Train" or "Exercise"  #3: [Title/Abstract] "cTn" or "Troponin"  #4: #1 and #2 and #3  Filters: Publication date from 2010/01/01 to 2022/6/23 | 476,274  464,918  20,394  101 |
| Web of Science | #1: TOPIC: ("Interval " or "Intermittent")  #2: TOPIC: ("Training" or "Train" or "Exercise")  #3: TOPIC: ("cTn" or "Troponin")  #4: #1 and #2 and #3  Refined by: PUBLICATION YEARS: (20220623-20100101)  Indexes=SCI-EXPANDED, SSCI, CCR-EXPANDED, | 650,520  1,034,315  27,362  114 |
| EBSCO | #1: Abstract: ("Interval " or "Intermittent")  #2: Abstract: ("Training" or "Train" or "Exercise")  #3: Abstract: ("cTn" or "Troponin")  #4: #1 and #2 and #3  Year: 20100101-20220623 | 835,462  1,591,635  26,489  117 |
