# Supplementary material for: Effects of high-intensity interval exercise on cardiac troponin elevation when comparing with moderate-intensity continuous exercise: a systematic review and meta-analysis
Source: PeerJ. 2023 Jan 11;11:e14508. doi: 10.7717/peerj.14508 (PMC9840388; doi:10.7717/peerj.14508)
Supplement: Supplemental Information 3 [file peerj-11-14508-s003.docx]

Purpose/Rationale of the systematic review

Exercise recovery mode may potentially affect the release of cardiac troponin after exercise. HIIE with passive recovery induced more cardiac troponin elevation after exercise compared to MICE. In clinical practice, this finding offers a valuable insight in the evaluation of raised cardiac troponins in patients who presented with angina equivalence symptom especially following exercise. This is particularly important for an acute care physician to differentiate a benign cause of raised cardiac troponin from myocardial infarction. Therefore, a serial troponin values would offer a more accurate diagnostic value and should be considered, in addition to the usual clinical evaluation and electrocardiogram.

This review aimed to explore the effects of recovery mode in HIIE versus MICE on the level of cardiac troponin after exercise. This information can help clinicians to estimate the outcomes of cardiac troponin after exercise, reduce misdiagnosis caused by exercise-induced cardiac troponin elevation, and help to establish safe HIIE exercise prescriptions.

The contribution that it makes to knowledge in light of previously published related reports, including other meta-analyses and systematic reviews.

Shave et al. ^31^ conducted a meta-analysis regarding the effects of exercise on cTn and found that compared with other athletes, endurance athletes released more cTn after exercise. A review by Cirer-Sastre et al.^32^ also indicated that the peak value of cTn might be related to the amount of exercise and the intensity of exercise.^32^ However, neither a systematic review nor meta-analysis has been performed regarding the effects of different recovery mode of HIIE on cTn.
