# Supplementary material for: Effects of high-intensity interval exercise on cardiac troponin elevation when comparing with moderate-intensity continuous exercise: a systematic review and meta-analysis
Source: PeerJ. 2023 Jan 11;11:e14508. doi: 10.7717/peerj.14508 (PMC9840388; doi:10.7717/peerj.14508)
Supplement: Supplemental Information 4 [file peerj-11-14508-s004.docx]

The formal screening was conducted after the review protocol was submitted to PROSPERO.

The protocol was submitted in March 2021, and published on 28 April 2021.

The delay in the registration was due to covid-19 pandemic, as stated in PROSPERO website.
